# The current status of dental graduates in India

**DOI:** 10.11604/pamj.2016.23.22.7381

**Published:** 2016-02-01

**Authors:** Sankalp Yadav, Gautam Rawal

**Affiliations:** 1 General Duty Medical Officer-II, Chest Clinic Moti Nagar, New Delhi, India; 2 Attending Consultant, Critical Care Department, Rockland Hospital, Qutab Institutional Area, New Delhi, India

**Keywords:** Dental graduates, government jobs, oral health

## Abstract

The dental profession is a noble profession. It takes years of devotion towards the subject of dentistry to get the graduate degree of Bachelor of Dental Surgery. However, even after such painstaking efforts the current situation of dental graduates in India is grave. There are a lot of issues that are the main cause for this problem. The dental graduates are in a state of crisis due to lack of support from the Government. If this situation continues it will lead to a negative effect on the integrity of the dental profession, and highly trained dental manpower of the country will go in vain.

## Commentary

The dental education came to light for the first time in India in 1920 when the first dental college was opened in Calcutta [1–3]. The first private dental college was established in 1966 [4]. Presently, there are 310 dental colleges in the country out of which 292 are privately owned and only 40 are run by the government [4–6]. There is a steep rise in the number of dental colleges all over the country. This rise in dental colleges has led to a higher number of dental graduates [6]. On one hand, this gradual increase in the number of dental graduates is good for the overall oral health of the country. However, on the other hand, there is growing number of dissatisfied dental graduates mainly because there are very low prospects of a job [4]. Records show that in 1970, there were only 8000 dental students graduated from Indian dental institutions, the figure was 30570 in the year 2010 [6]. This article highlights the grave situation of dental graduates in India and also suggests the solutions.

### Reasons for the current crisis

There are a lot of factors that have led to the present crisis in the dental graduates. Some of these factors could be associated with lack of interest in dental practice, random distribution of dental colleges all over the country, a lack of Government jobs, high competition in private practice, costly equipment's, lack of awareness about oral health in Indian public, very few postgraduate seats in Government colleges, very high fees for the private colleges, etc.

### Lack of interest in dental practice

The dental admissions resulting in bachelor of dental surgery (BDS) degree in India are through three ways [6]. State-administered entrance exams, common all-India entrance exams (for which all eligible students qualify) and private school-administered entrance exam [6]. The students who excel in these state-administered and common all-India entrance exams mostly take admissions in MBBS and admissions in BDS and other paramedical degrees, including nursing, physical therapy, pharmacy, and occupational therapy are usually the options available to lower ranks in the entrance [6]. Thus, most if not all, BDS admissions are not by choice and this leads to lack of interest in the dental practice [6].

### Uneven distribution of dental colleges

There are a number of dental colleges all over India. However, the distribution is uneven. A few states like Karnataka are having higher number of dental colleges as compared to some other states like Bihar, Gujarat, etc. [6]. Thereby, the students in such states have higher chances of pursuing BDS than those of states which are lacking such colleges [6]. Also, a number of private dental colleges are sub-standard and lack the basic infrastructure [6].

### Geographical factors

There has been a significant improvement in oral health over the years. However, this improvement has not been equal across the various sections of the population. The improvements in oral health of the urban population are better as compared to their rural counterparts. Thus, there is a significant mismatch between oral health professionals in India and the population that they serve. Most of the dentists are working in urban areas and those taking care of rural population are very few in number [2, 3, 5, 7–10]. As per the WHO, the provision of oral health care services is very little in rural parts of India, further complexity is lent by the great variation that occurs across this population on social parameters such as income and education [11]. Few of the studies conducted on the rural population of India have concluded that the unmet treatment need of the population is very high and the services present are inadequate in most parts of the country [11–14]. This higher concentration of dentists in the urban areas leads to competition and this competition may also lead to a number of social and behavioral issues in dentists, like involvement in unscrupulous and corrupt practices. Besides, very high competition can also take a toll on the mental and physical health of the dentists [4]. There is an imbalance between the demand and the supply of dental professionals [4]. According to the WHO ideal Dentist-population ratio is 7500 [4]. In 2004, Dentist-population ratio in India was 1:30000. According to World Health Statistics - 2014, the ratio is 1:10000 [4]. In the year 2004, India had one dentist per 10000 people in urban areas and one dentist per 1.5 lakh people in the rural areas [15].

### Lack of Government jobs

The vacancies for dental professionals in Government sectors are also very less in number [4]. Records show that only 5% graduated dentists are working in the government sector [4, 6]. Besides, the salaries in these Government hospitals vary a lot among various parts of the country. Also, the selection procedure for such posts may also be overshadowed by the growing corruption and malpractices.

### Difficulties related to private practice

Most of the dentists also resort to private practice in their own clinics. But opening a private setup requires a healthy investment. The cost of equipment's and the locality of clinics need sound financial support. Even after such financial constraints the private practice is not easy due to already saturated market and competition [4]. Also, the lack of awareness about oral health forces such clinics to be concentrated in the urban areas and not in rural areas where these clinics are usually not successful.

### Less number of post-graduate seats

The dental graduates are facing serious problems. The number of postgraduate seats in the Government colleges are also very few. The dental graduates have to face a lot of competition to get admission to the postgraduate colleges. A survey at a Government dental college showed that about 40% first year and about 70% fourth year dental students are interested in pursuing post-graduation [6]. Although, the private colleges are there in the education sector that offer post-graduation in various dental disciplines, but these colleges charge a hefty fee and are not in the reach of most of the dental graduates. Jain et al. 2012 mentioned that the total number of post-graduate seats available is only around 3000 compared to each year pass outs of 25000 dental graduates [6]. The above mentioned problems are just a glimpse of the hardships faced by the dental graduates. The lack of proper job, absence of post-graduate education and endless competition in the market leads to mild to severe mental health issues. Thereby, the psychological health of dentists is in jeopardy. There are many cases of depression leading to suicides amongst dental graduates [4]. Besides, many dentists resorts to other professions which are usually sub-standard or they may also resort to cheap antisocial practices [4].

### What can be done?

A number of papers are available in medical literature highlighting the problems of dentists in India, but still no radical steps are taken by the Government of India to improve the situation of the dental graduates. A number of steps are essential to control the poor situation of dental graduates. Firstly, there should be a control over the number of dental colleges as there are a higher number of dental colleges than the total number of post-graduate seats for dental graduates. It is high time to control oversupply of dental manpower as it is leading only to higher unemployment rates. If the present situation continues, there will be more than 1 lakh dentist's oversupply by the year 2020 [2]. Also, the Dental Council of India (DCI) should take strict actions over those dental colleges that are not up to the standards or are not as per the DCI guidelines [4]. The DCI should conduct a single uniform entrance exam of dentistry independent of medical entrance examinations. And this dental entrance exam should not be linked to the medical admission exam [6]. This way only those candidates who are really interested in the dental profession will apply for admission [6]. DCI can follow the American system of the Dental Admission Test (DAT); a single entrance test exists for all dental colleges [6]. The number of post-graduate dental seats should be increased. This will give greater opportunities to the dental graduates to pursue post-graduation.

The imbalance between the rural and urban dentists can be improved by increasing job opportunities in rural areas, thereby the rural areas will attract dental graduates and thus the concentration of dental graduates in the urban areas will diverge to underserved areas. The Government of India is taking an initiative to set up dental practices in rural areas by providing the subsidies, which is really essential [6]. The Government of India should plan to create new posts for dental graduates in government hospitals and at the Primary Health Centers [4]. Oral health programs should be planned to provide dental health education to increase oral health awareness especially among the rural population [4]. The inadequacy in primary care services for oral health is also highlighted in Universal Health Coverage report of the Planning Commission of India, which may affect the India's ambition to have universal health coverage [5]. The present situation of dental graduates is critical and requires radical decisions to be taken. The Indian healthcare industry is growing very fast owing to the increasing demand for quality healthcare. However, even in such situation the condition of dental graduates is not improving. Factors like, non-uniformity of admission procedure, uneven distribution of dental colleges across India, decreased quality of education and hurdles to establish a private practice, difficulty in getting post-graduation, financial security, etc. are some of the reasons that play main role in such crisis. Although all of these factors cannot be eliminated immediately, but DCI and the Government of India should take steps to retain the interest of dental graduates within the dental stream. If appropriate decisions are not made on time, it will negatively affect the integrity of the dental profession, and highly trained dental manpower of the country will go in vain.
